# MMP-14 overexpression correlates with the neurodegenerative process in familial amyloidotic polyneuropathy

**DOI:** 10.1242/dmm.028571

**Published:** 2017-10-01

**Authors:** Diana Martins, João Moreira, Nádia Pereira Gonçalves, Maria João Saraiva

**Affiliations:** 1 Instituto de Inovação e Investigação em Saúde (I3S), Universidade do Porto, R. Alfredo Allen 208, 4200-135 Porto, Portugal; 2 Neurobiologia Molecular - Instituto de Biologia Molecular (IBMC), Universidade do Porto, R. Alfredo Allen 208, 4200-135 Porto, Portugal

**Keywords:** MMP-14, Familial amyloidotic polyneuropathy, Neurodegeneration, Neuroinflammation, Peripheral nervous system

## Abstract

Levels of matrix metalloproteases (MMPs) can be differentially regulated in response to injury or neurological diseases. For instance, it is known that selective and short-term inhibition of MMP-14, a membrane-type 1 MMP, accelerates axon regeneration. Because axon growth and regeneration is impaired in familial amyloidotic polyneuropathy (FAP), a neurodegenerative disorder characterized by misfolding and deposition of mutant transthyretin (TTR) in the peripheral nervous system (PNS), we presently investigated the expression levels and the potential role for MMP-14 in this condition. By using cell culture studies, a mouse model of disease and human clinical samples, we observed that MMP-14: (i) is overexpressed in FAP nerves, correlating with TTR deposition; (ii) is upregulated in sciatic nerves from a preclinical transgenic mouse model, increasing with TTR deposition; (iii) levels in the PNS and plasma are rescued upon treatment of mice with anakinra or *TTR* siRNA, drugs acting over the IL-1 signaling pathway or TTR liver synthesis, respectively; (iv) increases in Schwann cells upon incubation with amyloid-like aggregates; and, finally, (v) is increased in plasma of FAP patients, correlating with disease progression. These results highlight the relevance of MMP-14 in the pathophysiology of FAP, suggesting not only a potential role for this molecule as a novel biomarker for therapy follow up, but also as a new potential therapeutic target.

## Abstract (precis)

**Summary:** MMP-14 overexpression correlates with familial amyloidotic polyneuropathy disease and progression, as determined using mouse models and human samples. *TTR* gene silencing in the preclinical model decreases MMP-14 plasma levels.

## INTRODUCTION

Transthyretin (TTR)-related amyloidosis is the most common form of hereditary autosomic dominant systemic amyloidoses; TTR point mutations lead to the extracellular deposition of amyloidogenic species in different tissues (Benson and Kincaid, 2007). A variety of TTR mutations are known; however, the most common mutation related to peripheral neuropathy is TTR V30M, which represents the replacement of a methione for a valine at codon 30 (Saraiva et al., 1984). Familial amyloidotic polyneuropathy (FAP) is associated with deposition of mutant TTR aggregates and amyloid fibrils, particularly in the peripheral nervous system (PNS), accumulating in the basal lamina of Schwann cells, blood vessels and collagen fibrils, ultimately leading to axonal fiber degeneration and cell death (Coimbra and Andrade, 1971; Planté-Bordeneuve and Said, 2011). Recently, TTR expression was found in Schwann cells of the sciatic nerve (Murakami et al., 2015), but this protein is mainly synthesized in the liver and choroid plexus of the brain (Soprano et al., 1985), being secreted into the blood stream and cerebrospinal fluid, respectively.

Although the mechanisms leading to TTR deposition comprise an alteration in the conformational stability of the homotetrameric form, followed by the deposition of unfolded monomers, aggregates or amyloid fibers (Quintas et al., 2001), the mechanisms mediating cytotoxicity in FAP are not completely understood. Several molecular pathways, such as oxidative stress, apoptosis, the heat-shock response and extracellular matrix remodeling, have been implicated in the pathogenesis of FAP (Saraiva et al., 2012). Inflammation has an important role not only as a causing agent but also in TTR deposition and consequently neurodegeneration (Sousa et al., 2001a,b; Buxbaum et al., 2012; Gonçalves et al., 2014a). Also, production of regeneration mediators, such as chemokines and neurotrophins, by Schwann cells is deficient in FAP nerves (Sousa et al., 2001a,b; Gonçalves et al., 2014b). Therefore, the disclosure of novel mechanisms and disease modifying agents underlying nerve degeneration is essential to understand disease progression. In this regard, work on high-throughput gene arrays has shown that matrix metalloproteinase-9 (MMP-9) was increased in FAP and is able to *in vitro* degrade TTR aggregates and fibrils (Sousa et al., 2005). Moreover, MMP-9 is transcriptionally upregulated by NF-κB, a transcription factor that is activated in FAP nerves.

Given the relationship between inflammation and extracellular matrix (ECM) remodeling, and the increase of proinflammatory cytokines in FAP, we further search for misexpression of other MMPs in FAP peripheral nerves. MMPs are zinc- and calcium-dependent endopeptidases, identified as matrix-degrading enzymes (Candelario-Jalil et al., 2009), and are members of an extracellular protease family of collagenases, gelatinases, stromelysins and membrane-type MMPs (Liu et al., 2010). The regulation of the ECM by proteases is a fundamental biological process for normal growth, development and repair (Candelario-Jalil et al., 2009). In normal conditions, the highly coordinate action of these enzymes remodel the components of the matrix and perform essential functions at the cell surface involved in signaling and cell survival.

The membrane-type 1 matrix metalloproteinase (MMP-14) is a pro-tumorigenic factor: its upregulation is associated with glioma expansion (Markovic et al., 2009; Langenfurth et al., 2014). Moreover, MMP-14 expression is found in microglia/macrophages associated with neurodegenerative and neuroinflammatory pathologies both in mouse models and human biopsies (Langenfurth et al., 2014). In patients with Alzheimer's disease (AD), MMP-14 was found overexpressed in brain (Yamada et al., 1995), which was later corroborated by studies using a transgenic mouse model where MMP-14 was found in reactive astrocytes, in regions with fibrillar amyloid deposits (Liao and Van Nostrand, 2010). More recently, the importance of MMP-14 in the PNS was noted to be related to its association with neuronal glial antigen 2 (NG2) proteoglycan on macrophages and further coordination of the response to peripheral nerve injury (Nishihara et al., 2015).

Based on the above, in the present study, we investigated the expression levels of MMP-14 in tissues from FAP patients carrying the TTR V30M mutation and in a disease mouse model to understand whether this molecule could function as a new marker for neurodegeneration in the pathogenesis of FAP.

## RESULTS

### Overexpression of MMP-14 correlates with TTR non-fibrillar deposition in the nerve of a FAP mouse model

**Fig. 1.**
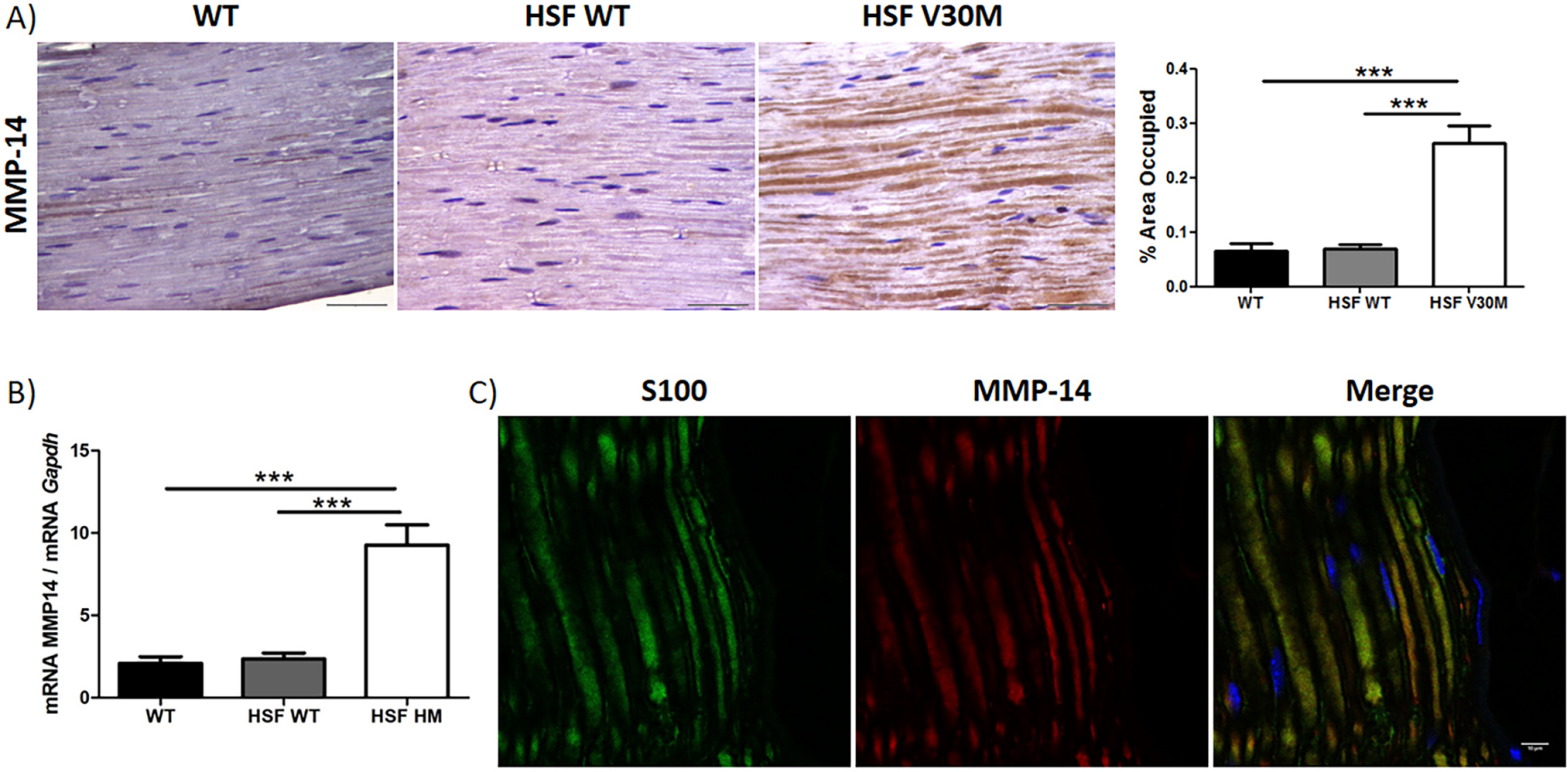
**Overexpression of MMP-14 in a naïve FAP mouse model.** (A) MMP-14 protein levels in the peripheral nerve system (*n*=6) assessed by semi-quantitative IHC, with respective semi-quantification (right-hand chart) presented as mean±s.e.m. (***P<0.001), demonstrating an increasing expression of MMP-14 in sciatic nerve of Hsf/V30M mice (scale bars: 100 µm). (B) Upregulation of MMP-14 in the sciatic nerve of Hsf/V30M mice (*n*=6) evaluated by qPCR (***P<0.001), compared with WT (*n*=6) and Hsf/WT (*n*=6) mice. Data were analyzed using one-way ANOVA followed by Bonferroni post-test and represented as mean±s.e.m. Normalization was performed against *Gapdh* mRNA. (C) Confocal microscopy of transgenic peripheral nerve showing immunopositive staining of MMP-14 and Schwann cells, as highlighted by colocalization with S100 (scale bar: 10 μm).

We started by analyzing MMP-14 expression in sciatic nerve from 6-month-old Hsf/V30M mice (*Hsf-1*-deficient mice transgenic for human TTR V30M, in the Sv/129 and endogenous *Ttr*-null background) by both immunohistochemistry (IHC) and quantitative real-time polymerase chain reaction (qPCR). Significant overexpression of MMP-14 protein and gene levels were observed when compared to age-matched control mouse strains, i.e. non-transgenic wild-type (WT; Sv129 strain) or Hsf-1-deficient mice (Fig. 1A,B). In both control and transgenic animals, MMP-14 staining was observed at background levels in other tissues, with the exception of a few dorsal root ganglia (DRGs), and the difference in the level of staining between control and transgenic animals was not statistically significant (not shown). MMP-14 immunopositive co-staining in transgenic peripheral nerve was confirmed with double immunofluorescence labeling between this molecule and S100 (Fig. 1C); however, co-staining between MMP-14 and βIII-tubulin did not occur (not shown), indicating that MMP-14 is localized in Schwann cells.

To evaluate whether increased levels of MMP-14 are associated with TTR non-fibrillar deposition, we subsequently studied levels of this MMP protein in 3-, 6- and 22-month-old Hsf/V30M mice. Based on the results, we could perceive that younger animals have the lowest levels of MMP-14, which increased with age (Fig. S1), accompanying the increased nerve TTR deposition (Santos et al., 2010), thus suggesting an association between MMP-14 upregulation with the progress of the neuropathological features of FAP disease.

### Treatment of transgenic mice with anakinra or *TTR* siRNA rescued MMP-14 nerve protein levels

**Fig. 2.**
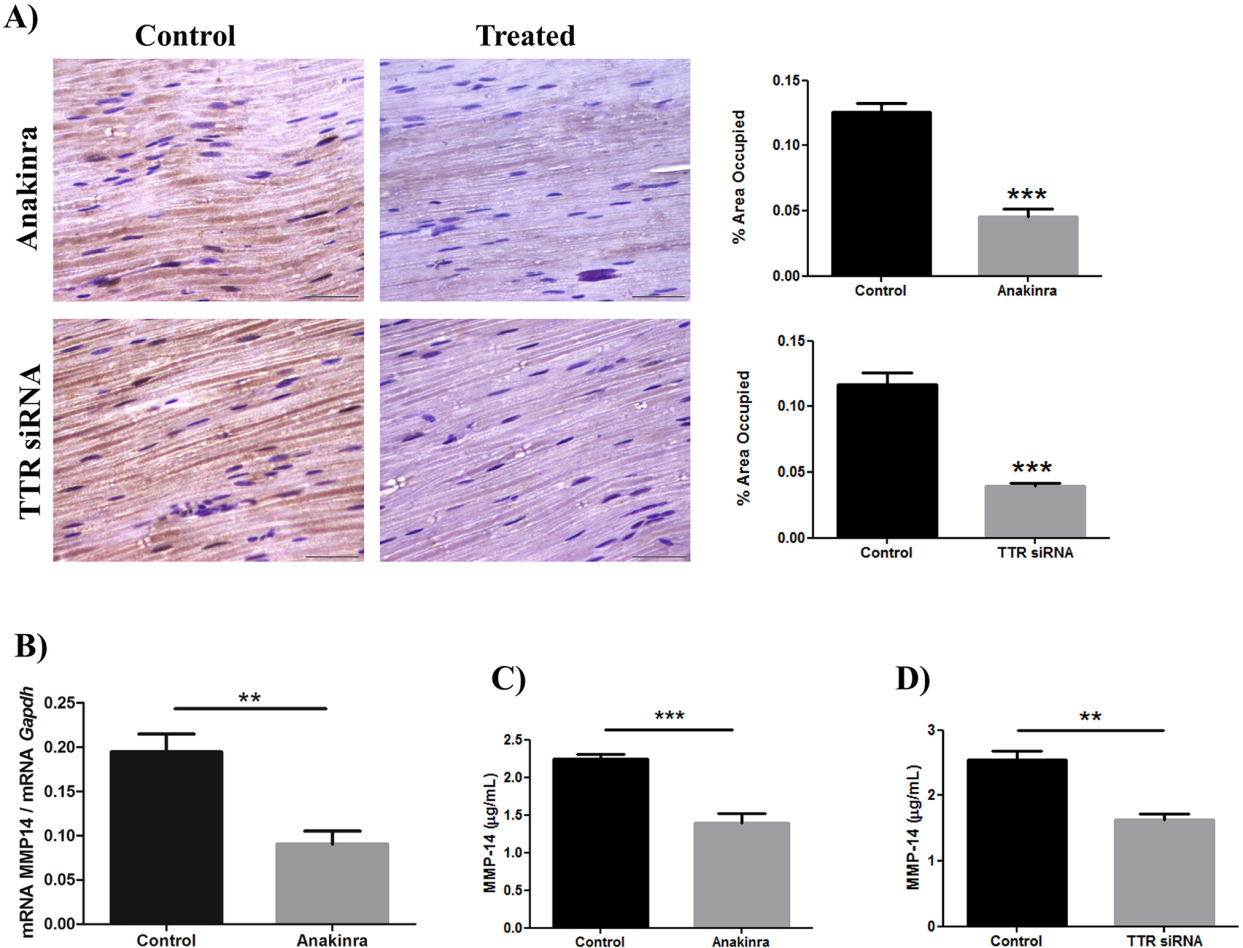
**Treatments with anakinra or *TTR* siRNA reduce MMP-14 expression in Hsf/V30M animals.** (A) Representative images obtained with IHC for MMP-14 using the Hsf/V30M mouse model, showing reduced MMP-14 expression in peripheral nerves of animals treated with anakinra or *TTR* siRNA. Charts represent quantification of the immunohistochemical images (scale bars: 100 µm) and data were analyzed using one-way ANOVA followed by Bonferroni post-test and represented as mean±s.e.m. (***P<0.001). (B) Downregulation of MMP-14 in the sciatic nerve of Hsf/V30M mice (*n*=6/group) evaluated by qPCR (**P<0.01), compared with mice treated with anakinra (*n*=6). Data were analyzed using one-way ANOVA followed by Bonferroni post-test and represented as mean±s.e.m. Normalization was performed against *Gapdh* mRNA. (C) Reduced levels of MMP-14, determined by ELISA, in sciatic nerves of mice treated with anakinra (*n*=6), compared with control mice (*n*=6). (D) Reduced levels of MMP-14, determined by ELISA, in sciatic nerves of mice treated with *TTR* siRNA (*n*=6), compared with control mice (*n*=6). Data were analyzed using one-way ANOVA followed by Bonferroni post-test and represented as mean±s.e.m. (**P<0.01; ***P<0.001).

Previous studies demonstrated that treatment of Hsf/V30M mice with anakinra or *TTR* siRNA prevents TTR non-fibrillar deposition in the peripheral nerve due to modulation of the inflammatory response or silencing liver *TTR* synthesis, respectively (Gonçalves et al., 2014b; Butler et al., 2016). To understand whether removal of nerve TTR deposits modulates the local synthesis of MMP-14, we performed IHC and qPCR for this molecule in nerves from treated and untreated animals. A significant statistical reduction of MMP-14 protein levels was observed, in both the anakinra- and siRNA-treated groups (Fig. 2A), results that were corroborated at the transcriptional level (Fig. 2B). In the context of being a novel biomarker for therapy efficacy, determination of MMP-14 levels in plasma are of major importance. Accordingly, measurement of MMP-14 protein levels in treated versus untreated animals denote a marked reduction of its levels in plasma after anakinra or *TTR*-siRNA treatment, possibly related to the reduction in TTR non-fibrillar deposition (Fig. 2C,D).

### Transgenic aggregated-TTR species induce expression of MMP-14 in Schwann cells

**Fig. 3.**
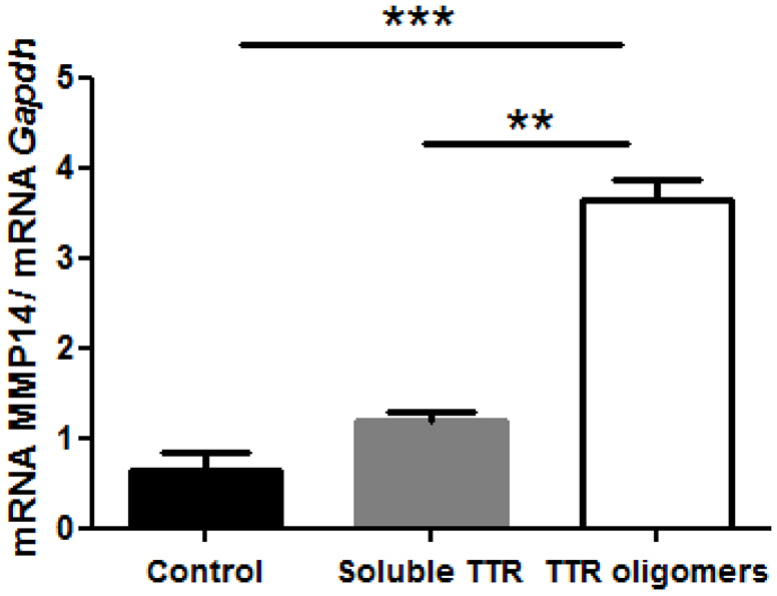
**MMP-14 expression is induced by aggregated-TTR species.** Upregulation of MMP-14 expression after incubation with Thioflavin-positive misfolded V30M aggregates (referred to in the chart as TTR oligomers) in mouse Schwann cells evaluated by qPCR, compared with non-treated cells or cells incubated with soluble TTR. Data were analyzed using one-way ANOVA followed by Bonferroni post-test and represented as mean±s.e.m. (**P<0.01; ***P<0.001).

Because MMP-14 expression in Schwann cells was previously described (Nishihara et al., 2015), we next performed an *in vitro* assay in order to determine whether aggregated-TTR toxic species could induce synthesis of MMP-14 in these glial cells. Therefore, TTR V30M aggregates were produced and incubated with primary mouse Schwann cells that were then lysed for mRNA extraction. Increased *MMP-14* mRNA was observed after 16 h incubation with TTR V30M oligomers, in contrast with levels in non-treated Schwann cells or cells incubated with soluble TTR V30M (Fig. 3).

### MMP-14 increases with the ongoing neurodegenerative process

**Fig. 4.**
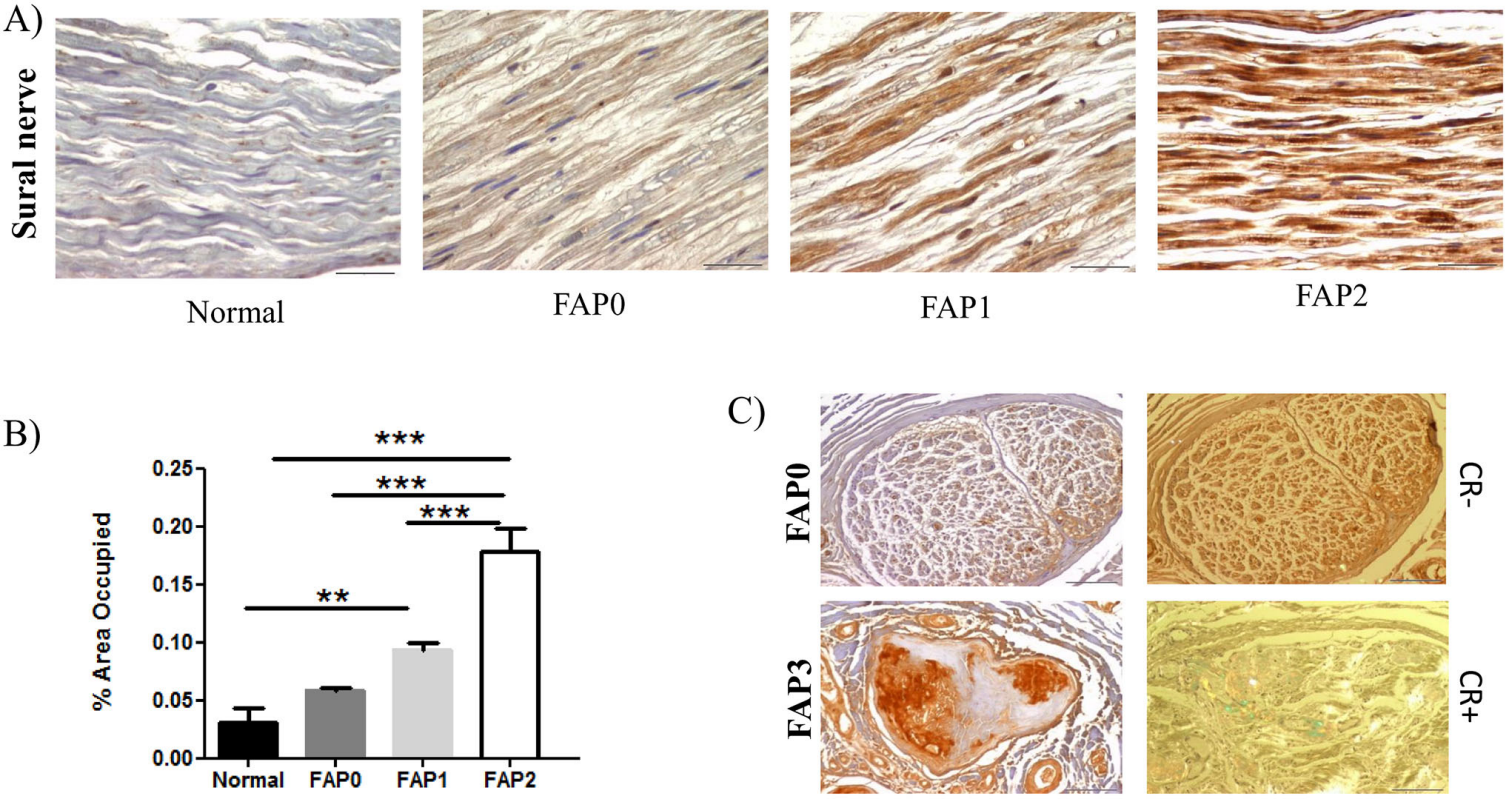
**MMP-14 expression in FAP patients at different stages of disease.** (A) Representative images obtained with IHC for MMP-14 in human sural nerve, showing an increased expression of MMP-14 in FAP patients in different stages of the disease, compared with asymptomatic and control individuals (*n*=6/group). (B) Chart represents quantification of the immunohistochemical images. Data were analyzed using one-way ANOVA followed by Bonferroni post-test and represented as mean±s.e.m. (**P<0.01; ***P<0.001). (C) Representative IHC images of MMP-14 expression and Congo Red (CR) staining showing association between MMP-14 expression and amyloid deposition (*n*=5/group). Scale bars: 100 µm.

Based on the nerve fiber density, a scoring system has been used for the classification of patient material and stage of disease. In this way, patients classified as FAP0 are asymptomatic, FAP1 subjects present a discrete decrease in nerve fiber density and fibrillar TTR depositions start to appear in the peripheral nerve, whereas individuals with FAP2 or 3 have evident or severe fiber reduction, respectively, with extensive amyloid deposition (Sousa et al., 2001a,b). To validate the *in vitro* and preclinical data described above, we next investigated MMP-14 expression in sural nerve biopsies from FAP patients in different stages of disease as compared with normal control subjects. IHC analyses demonstrated a significant increase in MMP-14 expression in nerve in advanced stages of disease, with non-fibrillar TTR and amyloid deposits being simultaneously present in the tissue (Fig. 4A,B). Asymptomatic carriers (FAP0) had no amyloid deposits in the sural nerve and low MMP-14 protein expression was observed. In contrast, at stage 3 (FAP3), amyloid deposition was heavily distributed throughout the tissue with massive neurodegeneration. Interestingly, MMP-14 seems to colocalize with amyloid deposits (Fig. 4C).

**Fig. 5.**
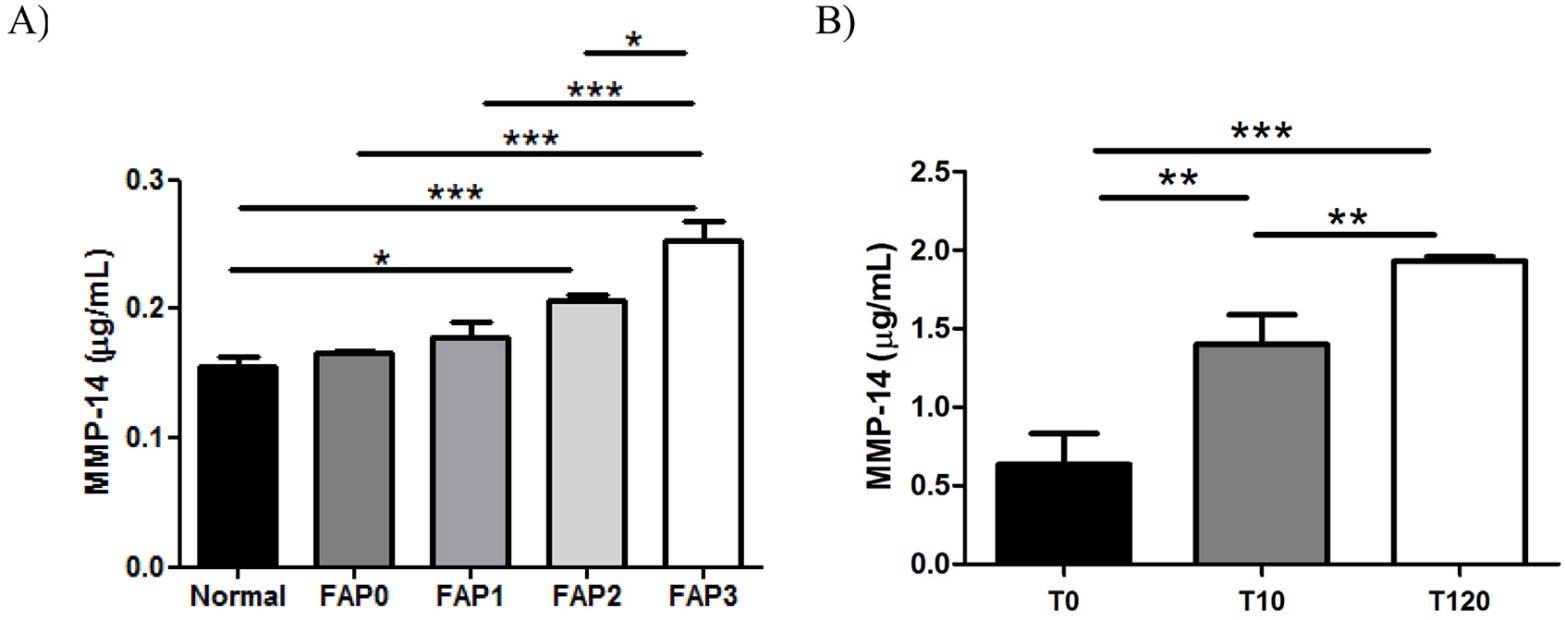
**MMP-14 levels, determined by ELISA, on plasma from human patients in different stages of disease.** (A) Increased levels of MMP-14 in FAP2 and FAP3 plasma samples, as compared to controls (*n*=5/group). (B) Increased plasma concentration levels of MMP-14 in patients transplanted with a FAP liver (10 and 120 days post-transplant), as compared with before transplant. Data were analyzed using one-way ANOVA followed by Bonferroni post-test and represented as mean±s.e.m. (*P<0.05; **P<0.01; ***P<0.001).

Additionally, we analyzed MMP-14 concentrations in plasma samples from patients in different stages of disease (as per the above criteria), asymptomatic carriers and normal individuals. We did not notice a difference in plasma levels between normal, asymptomatic and early stages of disease; however, a significant difference in MMP-14 levels was noted in advanced stages (Fig. 5A). Finally, we measured MMP-14 in plasma samples from individuals who received a liver transplantation from a donor with FAP (a FAP liver) as a form of treatment for severe liver conditions. We measured concentrations before transplantation, and at day 10 and 120 after domino liver transplantation (DLT). As illustrated in Fig. 5B, significant changes in MMP-14 plasma levels occurred between 10 and 120 days, as compared with pre-transplant, indicating that systemic molecular changes are taking place in liver recipient patients.

Taken together, these results suggest that MMP-14 might be a potential disease and therapeutic biomarker in TTR polyneuropathy.

## DISCUSSION

In the present study, we describe for the first time a significant association between MMP-14 and FAP, a degenerative peripheral neuropathy. We observed that MMP-14 was significantly overexpressed in degenerated FAP nerves presenting massive amyloid deposits and was also upregulated in sciatic nerves from a preclinical transgenic mouse model. Regulation of ECM is a fundamental biological process for normal growth, development and repair of the nervous system (Candelario-Jalil et al., 2009), enhancing the rate of sensory axonal regeneration by promoting Schwann cell mitosis (Liu et al., 2010), limiting the extent of myelin and axonal damage (Nishihara et al., 2015). Results from the present work suggest that major alterations in ECM-related genes occur in Schwann cells during FAP disease, such as MMP-14 misexpression. Our data also demonstrated that treatment with a *TTR* siRNA or anakinra significantly reduced MMP-14 expression in Hsf/V30M animals, both in peripheral nerves and plasma samples, pointing towards a role of both deposit clearance and inflammation in the modulation of MMP-14 expression. In fact, MMP-14 overexpression was also noticed in disorders with a strong neuroinflammatory component in their pathogenesis, such as multiple sclerosis or stroke, further pointing to a link between regulation of MMP-14 and neuroinflammation (Bar-Or et al., 2003; Langenfurth et al., 2014). Furthermore, increased levels of MMP-14 were also found in other amyloid-like neurodegenerative disorders such as Alzheimer's disease (AD). In AD patients, MMP-14 was found overexpressed in brain (Yamada et al., 1995), which was later corroborated by studies using a transgenic mouse model where MMP-14 was found in reactive astrocytes, in regions with fibrillar amyloid deposits (Liao and Van Nostrand, 2010). A common physiological mechanism involved in amyloidogenic disorders such as AD or FAP is the activation of inflammatory cascades (Sousa et al., 2001a,b). In FAP, the main inflammatory mechanism relies on, among others, the binding of amyloidogenic TTR species to the receptor for advanced glycation end products (RAGE) and, as a result, on activation of extracellular signal-regulated kinases 1/2 and NF-κB, with consequent transcription of pro-inflammatory proteins, such as IL-1β and TNF-α (tumor necrosis factor-α) (Monteiro et al., 2006; Sousa et al., 2000). Thus, it is possible that the increased levels of MMP-14 observed in neurological tissues result from the inflammatory process itself, also substantiated by the rescue upon treatment with anakinra. Further corroborating the interplay between inflammation and MMP-14 are recent data from Gao and colleagues (Gao et al., 2015), where an inhibition of MMPs, including MMP-14, accelerates wound healing in diabetic animals by decreasing inflammation.

MMP-14 is also responsible for pericellular remodeling events mediated by NG2 in the nervous system. Nishihara and colleagues showed that NG2 is expressed by macrophages and progenitor glia, and that NG2 shedding and axonal growth depends on MMP-14, highlighting the relevance of this MMP for axon regeneration, and pinpointing MMP-14 as a novel target in PNS injury and neuropathic pain (Nishihara et al., 2015). In preliminary experiments using macrophages or sciatic nerve from FAP transgenic mice, we did not confirm NG2 shedding in our model (data not shown). However, MMP-14 expression by macrophages might explain the entering of this molecule into the circulation and the augmented levels found in FAP plasma samples. Another possibility is that the increased MMP-14 produced by the Schwann cells could reach the blood stream through the blood-nerve barrier. Further corroborating this last hypothesis is the fact that MMP-14 was found increased in the plasma of patients even after liver transplantation, a likely consequence of nerve molecular changes.

MMP-14 can cleave other molecules of the ECM, such as fibronectin, laminin, cell adhesion molecules and even cytokines and growth factors (Ohuchi et al., 1997; Koshikawa et al., 2000; Kajita et al., 2001; Tam et al., 2004). Additionally, MMP-14 also enhances the proteolytic capacity through the activation of other MMPs, interfering directly with MMP-2 (Ishigaki et al., 1999; Langenfurth et al., 2014). In fact, it was previously described that MMP-14 is upregulated in glioma-associated microglia/macrophages through pro-MMP-2, suggesting MMP-14 as a protumorigenic factor (Markovic et al., 2009; Langerfurth et al., 2014). Interestingly, a previous study by our group has already shown an association between MMP-2 and ECM remodeling in FAP. In particular, the inhibition of *TTR* expression by an siRNA prevented and reversed TTR deposition, with a concomitant decrease on MMP-2 protein levels in DRG of a transgenic mouse line (Gonçalves et al., 2016). Based on our present results, it is possible to speculate that MMP-14 overproduced by FAP Schwann cells might consequently be transported to sensory neurons, contributing to the activation of MMP-2.

The cleavage of ECM components by MMPs, including MMP-14, can also originate damage-associated molecular patterns (DAMPs), which can interact with DAMP receptors, such as Toll-like receptors, amplifying the immune response to injury (Schaefer, 2014) and creating a feed-forward mechanism of injury in the course of FAP disease progression. Overall, we hypothesized that overexpression of MMP-14 in FAP might be associated with the inflammatory process and can also contribute to further remodeling of the ECM, for example by MMP-2 activation. Nevertheless, the specific role of MMP-14 in the pathophysiology of FAP remains unclear and warrants further investigation. So far, the demonstration of increased MMP-14 in the periphery, together with its augmented levels in plasma from FAP patients, especially in advanced stages of disease, denote a correlation between this MMP and neurodegeneration, highlighting its potential role as a novel disease biomarker or even as a promising future therapeutic target.

## MATERIALS AND METHODS

### Human samples

All human samples were collected from donors after written informed consent for the use of the material and clinical information for research purposes.

Formalin-fixed, paraffin-embedded sural nerve biopsy slides were obtained from FAP patients (*n*=3 for each disease stage), asymptomatic carriers (*n*=3) and matched disease controls (*n*=4) that were near-relatives of FAP patients who ultimately turned out not to have mutations in TTR. This material was kindly provided by the Hospital Geral de Santo António, Porto, Portugal and obtained as part of the clinical diagnosis, before the current use of less invasive methods, as previously described (Sousa et al., 2001a,b). The material was previously characterized, after informed consent, for TTR reactivity and amyloid deposition by histological analyses. Additionally, human plasma samples from control subjects, asymptomatic carriers and FAP patients in different stages of disease (*n*=5 for each group) were also available after informed consent. Plasma samples from individuals who had undergone DLT, hosting the liver of a FAP V30M patient, were available before DLT (*n*=5) and subsequently collected at different times after DLT (10 and 120 days; *n*=5 and *n*=2, respectively) after approval by the Ethics Committee of the Transplantation Department, University of Coimbra.

### Animals

All animal experiments were carried out in accordance with National and European Union guidelines for the care and handling of laboratory animals, and were performed in compliance with the institutional guidelines and recommendations of the Federation for Laboratory Animal Science Association (FELASA) and approved by the National Authority for Animal Health (DGAV; Lisbon, Portugal).

In the present study, transgenic mice for human TTR V30M, in the Sv/129 and endogenous *Ttr*-null background, with heterozygous deletion of the gene encoding transcription factor Hsf-1 (labeled as Hsf/V30M) (Santos et al., 2010) were analyzed at 3, 6 and 22 months of age (*n*=6 of each age). WT mice and WT animals heterozygous for *Hsf-1* deletion, both in the Sv/129 background, were used as controls (*n*=6 of each strain). Mice were housed in pathogen-free conditions in a temperature-controlled room and were maintained on a 12-h light/dark cycle, with water and food *ad libitum*. For animal sacrifice, a lethal injection of a premixed solution containing ketamine plus medetomidine was used. Plasma, sciatic nerve, DRG and other tissues typical of TTR deposition in FAP, such as the gastrointestinal tract, kidney and heart, were collected, as well as brain, liver and pancreas, representing tissues of TTR synthesis. They were frozen at −80°C, or fixed in formalin.

### Drug design and animal treatment

For anakinra (Kineret^®^, Biovitrum) treatment, Hsf/V30M mice (4.5 months of age) were daily injected subcutaneously with 25 mg anakinra/kg body weight over 6 weeks. Age-matched untreated control mice were injected with phosphate buffered saline (PBS). Because mice respond to anakinra in a dose range of 1 to 100 mg/kg (Abbate et al., 2008; Salloum et al., 2009), in this work we chose the intermediate dose of 25 mg/kg, as previously described (Gonçalves et al., 2014a).

For *TTR* siRNA treatment, Hsf/V30M mice (5 months) were injected in the tail vein with a human *TTR* siRNA (Semple et al., 2010), at a concentration of 1 mg/kg over 4 weeks. Untreated age-matched controls received vehicle only (*n*=4). In both groups, one intravenous injection was performed per week and animals were euthanized 48 h after the last injection.

### Semi-quantitative immunohistochemistry

Paraffin sections of human nerve and mouse sciatic nerve and sensory neurons were deparaffinized in Histoclear™ (National diagnostics, Atlanta, GA, catalog #HS-200) and hydrated. Endogenous peroxidase activity was blocked with 3% H_2_O_2_ in methanol for 30 min and sections were pretreated with blocking solution (10% fetal bovine serum and 0.5% Triton X-100 in PBS) to avoid undesired background staining. Sections were incubated overnight at 4°C with rabbit anti-MMP-14 (1:75, Millipore, Darmstadt, Germany, catalog #AB8345) in blocking buffer and, after washing, sections were incubated with the secondary biotinylated antibody horse anti-rabbit IgG (1:200, Vector Laboratories, Burlingame, CA, catalog #BA-1100). Avidin-biotin peroxidase complex (ABC Elite, Vector Laboratories, catalogb#PK-6100) labeling was performed with 3,3′-diaminobenzidine (Sigma, Darmstadt, Germany, catalog #D5637-1G) for antigen visualization. Sections were counterstained with hematoxylin (Millipore, catalog #1051752500) for 20 s, mounted using Entelan (Millipore, catalog#1079610500) and coverslipped for microscope visualization.

Semi-quantitative analysis of immunostaining was performed in 5 different areas at 20× magnification, representative of the whole tissue, using the Image pro-plus 5.1 software (Media Cybernetics, Rockville, MD). Quantification was blind. The results shown represent the percentage of occupied area by pixels corresponding to the substrate reaction color normalized relative to the total area occupied by the tissue, with the corresponding standard error of the mean (s.e.m.).

### Congo Red staining

The presence of amyloid deposits was investigated in human samples by Congo Red staining (Puchtler and Sweat, 1965). Briefly, deparaffinized tissue sections were incubated for 20 min with 0.01% NaOH in 80% ethanol saturated with NaCl followed by staining with 0.5% Congo Red in the previous solution. The slides were stained with hematoxylin and analyzed under polarized light. Amyloid was identified by the characteristic green birefringence.

### Confocal microscopy

Double immunofluorescence was performed to colocalize MMP-14 with Schwann cells (axons). After deparaffinazation, sections were hydrated and permeabilized with 0.5% Triton X-100 for 10 min and then blocked with phosphate buffer supplemented with 4% fetal bovine serum and 1% bovine serum albumin for 1 h at room temperature. After blocking, tissues were incubated overnight at 4°C with the primary antibodies: mouse monoclonal anti-βIII-tubulin (1:500, Promega), mouse monoclonal anti-S100 (1:100, Abcam), rabbit polyclonal anti-MMP-14 (1:75, Millipore). The following day, sections were incubated with secondary antibodies: Alexa-Fluor-488-conjugated goat anti-mouse IgG and goat Alexa-Fluor-568-conjugated anti-rabbit IgG (1:1000, Molecular Probes) for 2 h at room temperature. Finally, sections were mounted using Vectashield with 4′,6-diamino-2-phenylindole (DAPI; Vector Laboratories) for nuclei visualization. Image stacks were obtained using the laser scanning confocal microscope Leica TCS SP5 II (Leica Microsystems).

### Enzyme-linked immunosorbent assay

The levels of MMP-14 in human plasma of non-V30M-carrier controls (*n*=5), asymptomatic subjects (*n*=5) and FAP patients in different stages of disease (FAP1, FAP2 and FAP3) (*n*=5 per group) were quantitatively determined by enzyme-linked immunosorbent assay (ELISA), according to the manufacturer′s instructions (R&D Systems, Minneapolis, MN, catalog #DY918-05).

The levels of MMP-14 in plasma from Hsf/V30M mice before and after treatment with anakinra and *TTR* siRNA were also quantitatively determined, according to the manufacturers’ instructions.

### RNA extraction, cDNA synthesis and qPCR

RNA from sciatic nerves was isolated with RNeasy Mini columns, following the manufacturer′s instructions (Qiagen, Hilden, Germany, catalog #74804). cDNA synthesis was performed using the SuperScript II reverse transcriptase (Invitrogen, Waltham, MA, catalog #18064014). The primers to target MMP-14 were designed with the Beacon Designer 8 software (Premier Biosoft International) and the MMP-14 mouse forward 5′-TTACAAGTGACAGGCAAGG-3′ and mouse MMP-14 reverse 5′-GCTTCCTCCGAACATTGG-3′. A standard calibration curve was performed by 10-fold serial cDNA dilutions, to assess primer efficiency. qPCR was performed in duplicates using an IQ5 multi-color real-time detection system with IQ Sybr Green Super Mix and gene expression analysis performed with Bio-Rad iQ5 software (Bio-Rad Laboratories, Hercules, CA, catalog #1708880). Melting curves were produced in order to detect non-specific amplification products. Five biological replicates were used per group. Differential expression was determined by the 2^−ΔΔCT^ method, using the *Gapdh* gene for normalization.

### Preparation of TTR V30M aggregates

Recombinant TTR V30M was produced in *Escherichia coli* BL21 and purified (Furuya et al., 1991). Soluble TTR V30M was filtered through 0.2 μm Anotop syringe filters (Whatman, England) and TTR aggregates generated by incubating the protein (2 mg/ml), with stirring, at room temperature for 7 days (Teixeira et al., 2006). TTR preparations were then tested by Thioflavin T spectrofluorometric assay to search for their amyloidogenic potential. Dynamic light scattering (DLS) at 25°C in a Malvern Zetasizer Nano ZS (Malvern, Worcestershire, UK) was also performed to confirm TTR pathogenic aggregation and specimen size (Ferreira et al., 2011).

### Schwann cell primary culture

Mouse Schwann cell primary cultures were obtained from ScienCell Research Laboratories™ (San Diego, CA, catalog #M1700) and were cultured according to the manufacturer's instructions. Briefly, cells were cultured in Schwann cell medium, supplemented with 5% fetal bovine serum, 1% Schwann cell growth supplement and 1% penicillin/streptomycin (all from ScienCell Research Laboratories). After monolayer propagation in T-75 flasks at 37°C in a 5% CO_2_ humidified chamber, 5×10^5^ cells were seeded in 12-well plates and stimulated with TTR aggregates or soluble protein, both at 2.5 μM in duplicates. Twenty-four hours after stimulation, cell lysates were prepared in trizol (Invitrogen, Waltham, MA, catalog #15596026) and assayed for the expression of *MMP-14* mRNA by qPCR. Unstimulated cells were also used as controls.

### Statistical analysis

Statistical comparison of data was performed using Student's *t*-test or one-way analysis of variance (ANOVA) followed by Bonferroni post-test, with GraphPad Prism software. Quantitative data are expressed as mean±s.e.m. Statistical significance was established for *P<0.05, **P<0.01, ***P <0.001.

## Supplementary Material

Supplementary information
