# Supplementary material for: MMP-14 overexpression correlates with the neurodegenerative process in familial amyloidotic polyneuropathy
Source: Dis Model Mech. 2017 Oct 1;10(10):1253–60. doi: 10.1242/dmm.028571 (PMC5665453; doi:10.1242/dmm.028571)
Supplement: Supplementary information [file dmm-10-028571-s1.pdf]

A)

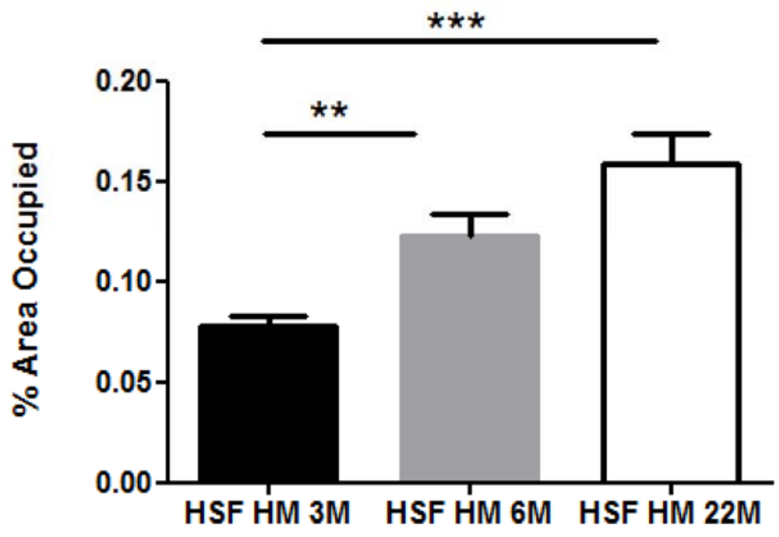

Supplemental Figure 1: MMP-14 upregulation and non-fibrilar TTR deposition in a naïve mouse model. **A)** Representative images obtained with IHC for MMP-14 using Hsf/V30M mouse model with 3, 6 and 22 months of age (n=6), showing an increase of MMP-14 expression in animals with 22 months. Chart represents quantification of immunohistochemical images (scale bar 100µm) and data was analyzed using one-way ANOVA followed by Bonferroni post-test and represented as mean± SEM (*** $p < 0.001$).
